# Distinctive effect of anesthetics on the effect of limb remote ischemic postconditioning following ischemic stroke

**DOI:** 10.1371/journal.pone.0227624

**Published:** 2020-01-16

**Authors:** Gangling Chen, Pradip Kumar Kamat, Abdullah Shafique Ahmad, Sylvain Doré

**Affiliations:** 1 Department of Anesthesiology, Center for Translational Research in Neurodegenerative Disease, McKnight Brain Institute, University of Florida, Gainesville, FL, United States of America; 2 Department of Pharmacology of Chinese Materia Medica, School of Traditional Chinese Pharmacy, China Pharmaceutical University, Nanjing, China; 3 Departments of Neurology, Psychiatry, Pharmaceutics, and Neuroscience, University of Florida, Gainesville, FL, United States of America; Massachusetts General Hospital/Harvard Medical School, UNITED STATES

## Abstract

Limb remote ischemic postconditioning (LRIP) has been reported as an effective method to reduce the induced experimental stroke damage after ischemic reperfusion (IR) injury. Studies suggest that anesthetics used during induction of ischemic stroke can reduce IR injury, which could affect the actual mechanisms of neuroprotection by LRIP. This study focuses on the comparative effects of anesthetics such as isoflurane and ketamine-xylazine on ischemic injury when used during LRIP. Adult C57BL/6 mice were anesthetized by isoflurane or halothane, and transient middle cerebral artery occlusion (MCAO) was induced through insertion of the filament. Under isoflurane or ketamine-xylazine anesthesia, LRIP was performed after 90 min of reperfusion by carrying out three cycles of 5 min ischemia/5 min reperfusion of the bilateral hind limbs for one session per day for a total of 3 days. Results showed that the use of different anesthetics—isoflurane or ketamine-xylazine—during LRIP had no effects on body weight. However, LRIP was able to improve neurological function as observed by the neurological deficit score in ischemic mice. Interestingly, the neurological deficit in the group where ketamine-xylazine was used was better than the group where isoflurane was used during LRIP. Furthermore, the LRIP was able to prolong the period of the ischemic mice on the rotarod and this effect was more significant in the groups where ketamine-xylazine was used during LRIP. Moreover, LRIP significantly attenuated the infarction volume; however, this effect was independent of the anesthetic used during LRIP. From these results, we conclude that ischemic mice that were subjected to LRIP under ketamine-xylazine anesthesia had better neurological deficit outcomes after stroke.

## Introduction

Cerebral ischemia, the most common acute cerebrovascular disease, has been recognized as one of the leading causes of mortality and disability in the world. Our previous studies have documented that either preconditioning or limb remote ischemic postconditioning (LRIP) is useful for reducing ischemic reperfusion (IR) brain injury [1,2], but the effects of common anesthetics on regulating stroke outcomes during middle cerebral artery occlusion (MCAO)-LRIP experiments have not yet been explored in detail. Anesthetics are widely used in experiments investigating neurotoxicity and neuroprotection; however, these agents are known to be able to interfere with experimental outcomes.

Some of the common anesthetics used during MCAO include chloral hydrate, isoflurane, sevoflurane, ketamine, and ketamine-xylazine [3–5]. These anesthetics have also been documented to have some anti-inflammatory [6,7] and anti-apoptotic properties [8], which may protect the brain from stroke pathology. Ketamine and halothane are also widely used anesthetics for many surgical procedures in animal models [9], and conflicting results have been reported. Ketamine has been shown to provide neuroprotective effects in rat models [9,10]. Ketamine is also reported to reduce brain injury by inhibiting apoptosis [11] and to attenuate IR in a chronic post-ischemic pain model of ischemia [7] and myocardial IR [6] through its anti-inflammatory effects. In another study, Ridenour *et al*. [12] used a rat MCAO model and showed that ketamine and halothane had no significant effects on cerebral infarct and neurological function. Ketamine has also been proved to reduce hind limb ischemia [7] and myocardium IR [6] through its anti-inflammatory effects. Other anesthetics used alone or in combination, such as sevoflurane and ketamine-xylazine, have also been proved to reduce injury in hepatic IR [13], cardiac IR [14], and brain IR [15].

Another common anesthetic, isoflurane, is widely used in many protocols and experiments such as LRIP [16] and brain IR [17]. Reports show that isoflurane was able to protect the heart and brain from ischemic damage [18–20]. Similarly, isoflurane was also found to reduce brain injury after MCAO in rodents [21] by reducing oxidative stress and inflammatory and apoptotic responses [22]. On the contrary, some reports also show that isoflurane had no effects on the final outcomes in an ischemic model. For example, Toner *et al*. (2002) induced *in vitro* ischemia using Wistar rat brain slices and reported that isoflurane does not provide cerebroprotective effects [23].

These studies indicate the potential of anesthetics to affect research outcomes. Moreover, the application of these anesthetics, along with the LRIP, may have confounding effects on the final research outcomes. Therefore, in this study, we tested the hypothesis of whether LRIP induction protects the brain from ischemic injury and how common anesthetics used during LRIP affect these outcomes. To test this hypothesis, we used isoflurane or halothane during MCAO, followed by isoflurane or ketamine-xylazine during LRIP.

## Materials and methods

### Animals

A total of 46 male wildtype (WT) C57BL/6 mice, aged 8–10 weeks old and weighing 26–30 g, were obtained from our Animal Care Services facility at the University of Florida. Using JMP Pro software (SAS Institute Inc.), we performed an *a priori* sample size calculation. For the computation of the required sample size, we used variances from our previous studies. To calculate effect size, we assumed that α = 0.05, standard deviation (SD) = 18, and power = 0.8. The study was powered with the expectation that we would detect a difference of at least 25% between the groups, which is a biologically meaningful effect [24,25]. The number of mice indicated below for each cohort also accounted for an anticipated mortality rate of ~20%. Mice were randomly assigned to four different cohorts: cohort 1: isoflurane MCAO + isoflurane sham LRIP (N = 12); cohort 2: isoflurane MCAO + isoflurane LRIP (N = 14); cohort 3: halothane MCAO + ketamine-xylazine sham LRIP (N = 9); cohort 4: halothane MCAO + ketamine-xylazine LRIP (N = 11). Mice were housed in our animal facility under controlled conditions (23 ± 2°C; 12h light/dark periods) and had access to food and water *ad libitum*. All experiments were approved by the University of Florida Institutional Animal Care and Use Committee under protocol #201605020. All animal experiments were performed according to the ARRIVE guidelines [26].

### Transient focal cerebral ischemia

MCAO surgery was performed as previously reported by our lab and other researchers [27,28]. Briefly, mice were anesthetized with isoflurane (4% induction and 1.5% maintenance) or halothane (3% induction and 1.0% maintenance). Between the eye and the ear, ~2 μl bupivacaine was applied topically, and an incision was made to fix a laser Doppler flowmeter (LDF) probe (Moor Instruments, Wilmington, DE) at the temporal bone over the MCA territory to measure cerebral blood flow (CBF). After that, ~2 μl bupivacaine was applied topically over the ventral side of the neck and an incision was made to isolate the common carotid artery (CCA), external carotid artery (ECA), and internal carotid artery (ICA). A 7–0 monofilament suture (Doccol Corporation, Sharon, MA) was inserted from the ECA to ICA, and the MCA was then occluded for 60 min. A successful occlusion was confirmed by a >80% drop in CBF as observed by LDF. The occlusion was further confirmed by mouse circling behavior during the occlusion phase. At 1 h of occlusion, reperfusion was initiated by withdrawing the monofilament, the incision was closed, and mice were returned to a recovery chamber. After that, mice were randomly divided into groups for LRIP treatment.

The body weight of the mice was assessed before MCAO and at the terminal endpoint at day 3. The animal health, postoperative care, and behavior were monitored daily until they were euthanized by decapitation under deep isoflurane anesthesia at day 3, the terminal time point of the study. Specific criteria for euthanasia before the terminal time point included hypothermia, seizure or barrel rolling, and inability to eat or drink even with assistance. However, with the exception of the mortality reported below, none of the mice reached these criteria; therefore, none were euthanized before the end of the experimental protocol. A total of five mice, two mice each from cohorts 3 and 4, and one mouse from cohort 1, died within 24 hours of surgery. These mice were found dead overnight and an analysis of the brain revealed massive infarction and edema. Three, one, and one mice from cohorts 2, 3, and 4, respectively, were excluded from the analysis following our preset exclusion criteria based on improper occlusion (CBF drop of < 80% from baseline), no circling during occlusion, or hemorrhage due to endoperforation by the monofilament. Thus, the total numbers of mice that were used in the analysis were 11, 11, 6, and 8 for cohorts 1, 2, 3, and 4, respectively.

### Limb remote ischemic postconditioning

**Fig 1:**
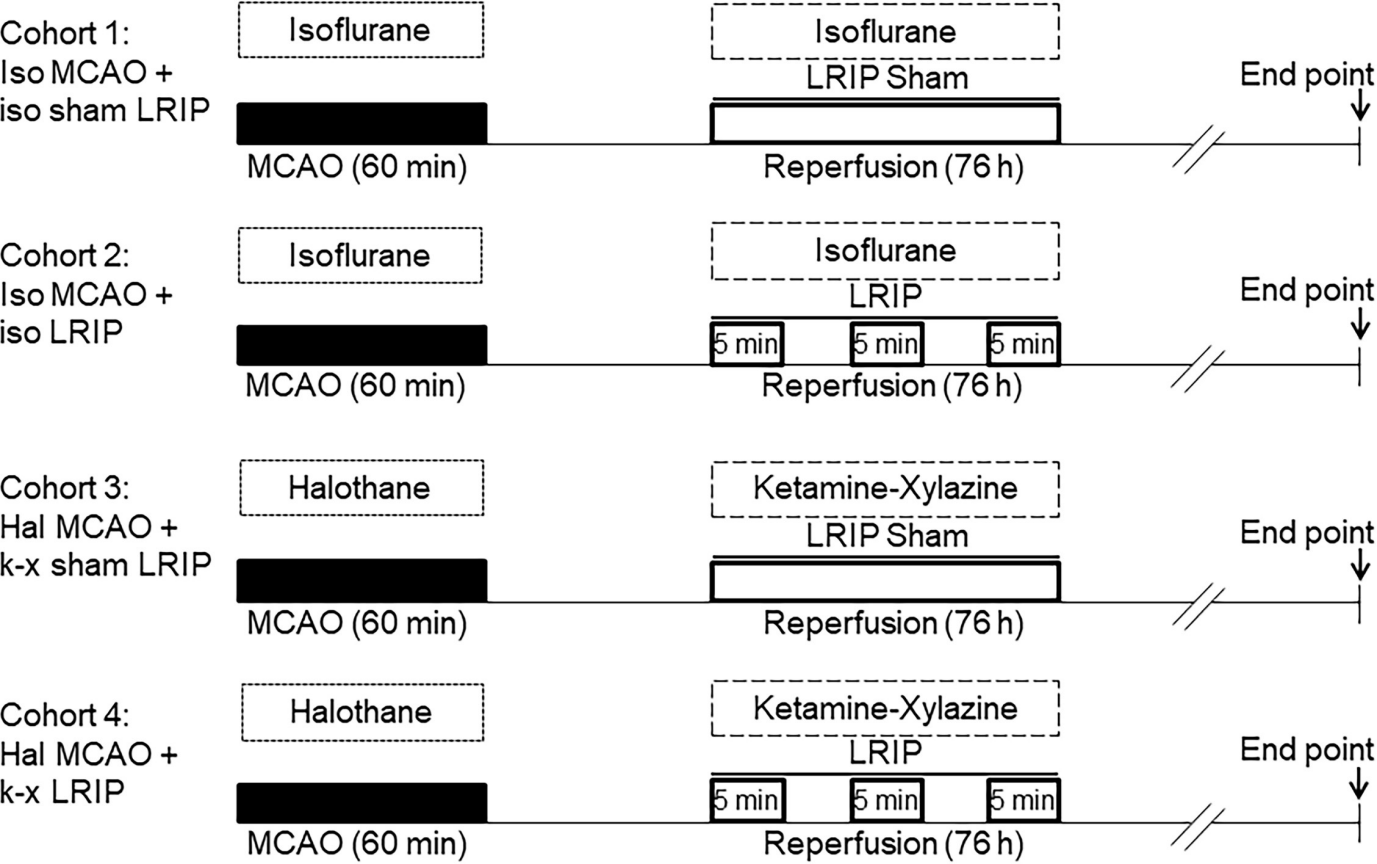
Experimental design. In cohort 1 (Iso MCAO + iso sham LRIP), mice underwent MCAO surgery under isoflurane anesthesia (Fig 1). At 90 min after reperfusion, sham-LRIP was induced under isoflurane anesthesia. In cohort 2 (Iso MCAO + iso LRIP), mice underwent MCAO surgery under isoflurane anesthesia. At 90 min after reperfusion, LRIP was induced under isoflurane anesthesia. In cohort 3 (Hal MCAO + k-x sham LRIP), mice underwent MCAO surgery under halothane anesthesia. At 90 min after reperfusion, sham-LRIP was induced under ketamine-xylazine anesthesia. In cohort 4 (Hal MCAO + k-x LRIP), mice underwent MCAO surgery under halothane anesthesia. At 90 min after reperfusion, LRIP was induced under ketamine-xylazine anesthesia. In cohorts 2 and 4, LRIP was induced at 90 min after reperfusion by three cycles of 5 min ischemia/5 min reperfusion of bilateral hind limb for one session per day for a total of four sessions.

The LRIP treatment was initiated at 90 min of reperfusion by using a CODA blood pressure measuring instrument (Kent Scientific, Torrington, CT). Briefly, to induce noninvasive limb ischemia, a medium-sized tail cuff was placed over both hind limbs. The cuff was inflated and the pressure was held for 5 min and then released to achieve reperfusion. Hind limb ischemia was induced for 5 min, followed by reperfusion for 5 min. This cycle was repeated 3 times/day for 3 days with an interval of 5 min between each cycle (4 sessions). Sham LRIP mice went through the same procedure except for the induction of hind limb ischemia. Mice were divided into four groups: cohort 1: isoflurane MCAO + isoflurane sham LRIP; cohort 2: isoflurane MCAO + isoflurane LRIP; cohort 3: halothane MCAO + ketamine-xylazine sham LRIP; cohort 4: halothane MCAO + ketamine-xylazine LRIP. In cohorts 1 and 2, MCAO was induced under isoflurane anesthesia followed by LRIP under isoflurane anesthesia. In cohorts 3 and 4, MCAO was induced under halothane anesthesia followed by LRIP under ketamine-xylazine anesthesia. A summary illustration of the experimental design is presented in Fig 1.

### Dosage of isoflurane, halothane, and ketamine-xylazine

The anesthetics and their dosages used in different experimental groups are isoflurane (4% induction and 1.5% maintenance), halothane (3% induction and 1.0% maintenance), ketamine, and xylazine (56 mg/kg and 8.75 mg/kg, respectively). Isoflurane and halothane were delivered by nose mask, whereas ketamine-xylazine were given in combination by intraperitoneal (i.p.) injection.

### Neurological deficit score (NDS)

The NDS was assessed at 24, 48, and 72 h post-MCAO surgery in a double-blind fashion by two investigators. We followed a 24-point neurological scoring system, as we described previously [29,30]. In brief, the test includes six parameters: body symmetry, gait, climbing, circling behavior, front-limb symmetry, and compulsory circling. Each of these parameters was given scores between 0 (no deficit) and 4 (extreme deficit). The scores of each parameter were then summed and the average of the sum obtained by two investigators was reported.

### Rotarod

This task was used to evaluate motor coordination. A Rotarod Rotamex 5 machine and software (Columbus Instruments, Columbus, OH) were used to measure latency to fall from the intermittently accelerating rotarod. Mice were pretested to collect baseline data prior to MCAO followed by testing at 24, 48, and 72 h after MCAO. Mice were placed on the rotarod moving at a constant speed of 4 rotation per min (rpm) with a constant acceleration at every 30 sec, reaching a top speed of 30 rpm over the course of 5 min, and the latency to fall from the rod was automatically recorded by the software. Every mouse was tested for three consecutive trials/day with 10-min intervals between each trial. The average times for all three trials were used for data analysis [31]. All testing was performed by investigators blinded to the experimental cohort and LRIP treatment.

### Infarct size measurement

At the terminal time point at day 3, all of the mice were euthanized by decapitation under deep isoflurane anesthesia and the brains were carefully removed. The brains were coronally sectioned into 2-mm slices, which were then incubated with 1% 2,3,5-triphenynyltrazolium chloride (TTC) and kept at 37°C for 20 min. After that, brain sections were fixed with 10% paraformaldehyde for 24 h. Images were captured with a digital scanner and then infarct areas were analyzed by using ImageJ software. The percent corrected infarct volume was calculated as: [volume of contralateral hemisphere–(volume of ipsilateral hemisphere–volume of infarct)/volume of contralateral hemisphere]*100 [32,33].

### Statistical analysis

Statistical significance was estimated by Student’s t-test for unpaired observations between two groups or by one-way analysis of variance (ANOVA) followed by Newman-Keuls multiple comparison tests. Results are considered statistically significant at *p* values < 0.05. For repeated measures of NDS and rotarod differences were analyzed by two-way ANOVA with subsequent group comparisons by non-parametric Wilcoxon test by using JMP Pro software. Data are expressed as means ± SD.

## Results

### Effect of LRIP using isoflurane or ketamine-xylazine on NDS in MCAO mouse

**Fig 2:**
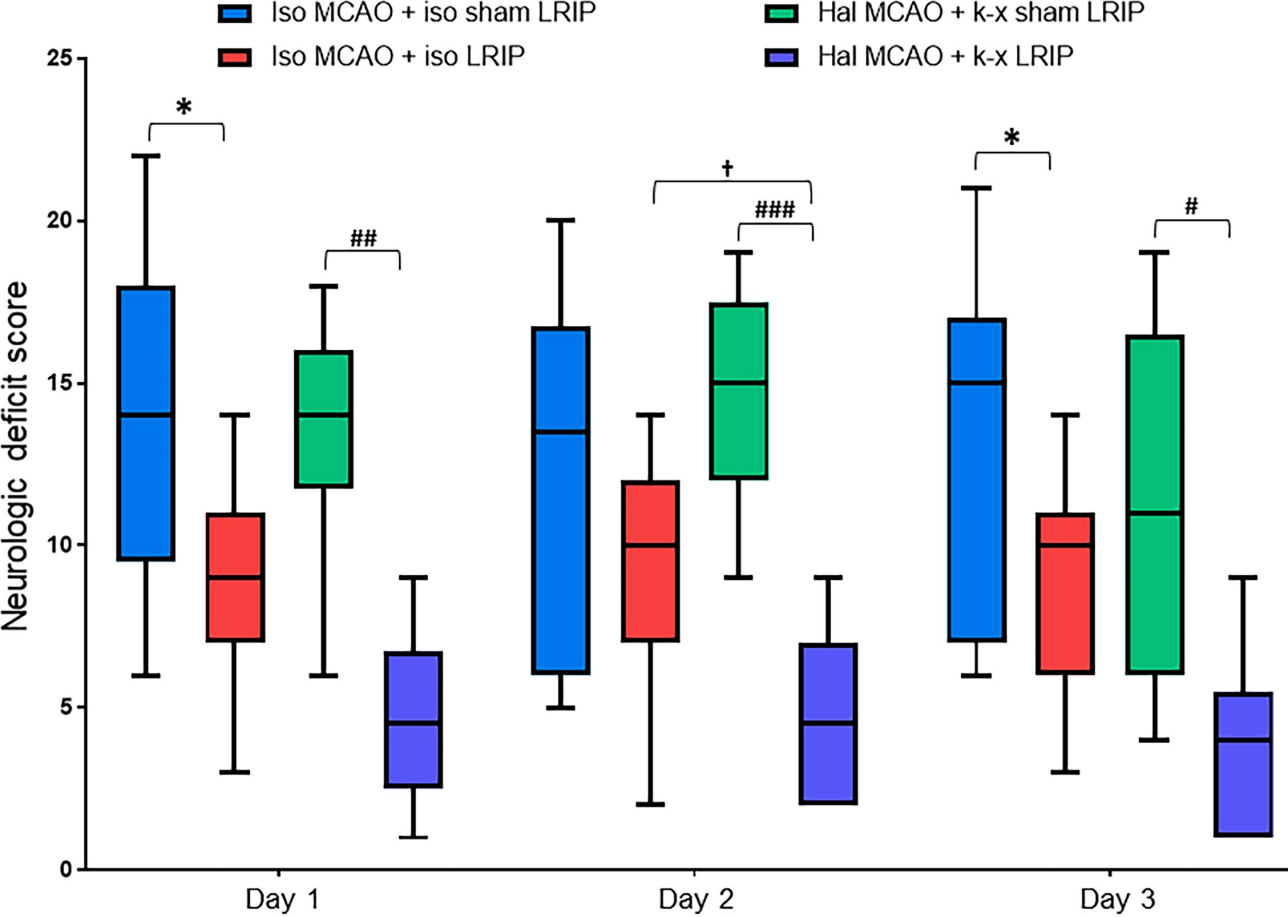
Effects of cerebral ischemia and LRIP on NDS. MCAO significantly augmented the neurologic deficit, which was significantly attenuated by LRIP treatment. Additionally, cohort 4, where ketamine-xylazine was used during LRIP, exhibited a significant difference compared with cohort 2, where isoflurane was used during LRIP. The effects of LRIP on NDS persisted across the time points. Data are presented as mean ± SD. *p < 0.05 when compared cohort 1 vs cohort 2, ^#^p < 0.05, ^##^p < 0.01, ^###^p < 0.001 when compared cohort 3 vs cohort 4, and ^†^p < 0.05 when compared cohort 2 vs cohort 4.

After MCAO, the body weights of mice from each experimental group decreased from day 1 to day 3; however, no significant difference was observed between the groups. For NDS, in the comparison between cohorts 1 and cohort 2 that used isoflurane during MCAO as well as during LRIP, a significant (p < 0.05) improvement was observed in cohort 2 on day 1 and day 3. The comparison between cohort 3 and cohort 4 that used halothane during MCAO and ketamine-xylazine during LRIP showed significant improvement in NDS in cohort 4 at all post-MCAO time points (p < 0.01 at day 1, p < 0.001 at day 2, and p < 0.05 at day 3). Of interest, the comparison of cohort 2 (group with isoflurane during LRIP) and cohort 4 (group with ketamine-xylazine during LRIP) revealed a significantly (p < 0.05) improved NDS in cohort 4 at day 2 (Fig 2).

### Effect of LRIP using isoflurane or ketamine-xylazine on the rotarod scores in MCAO mice

**Fig 3:**
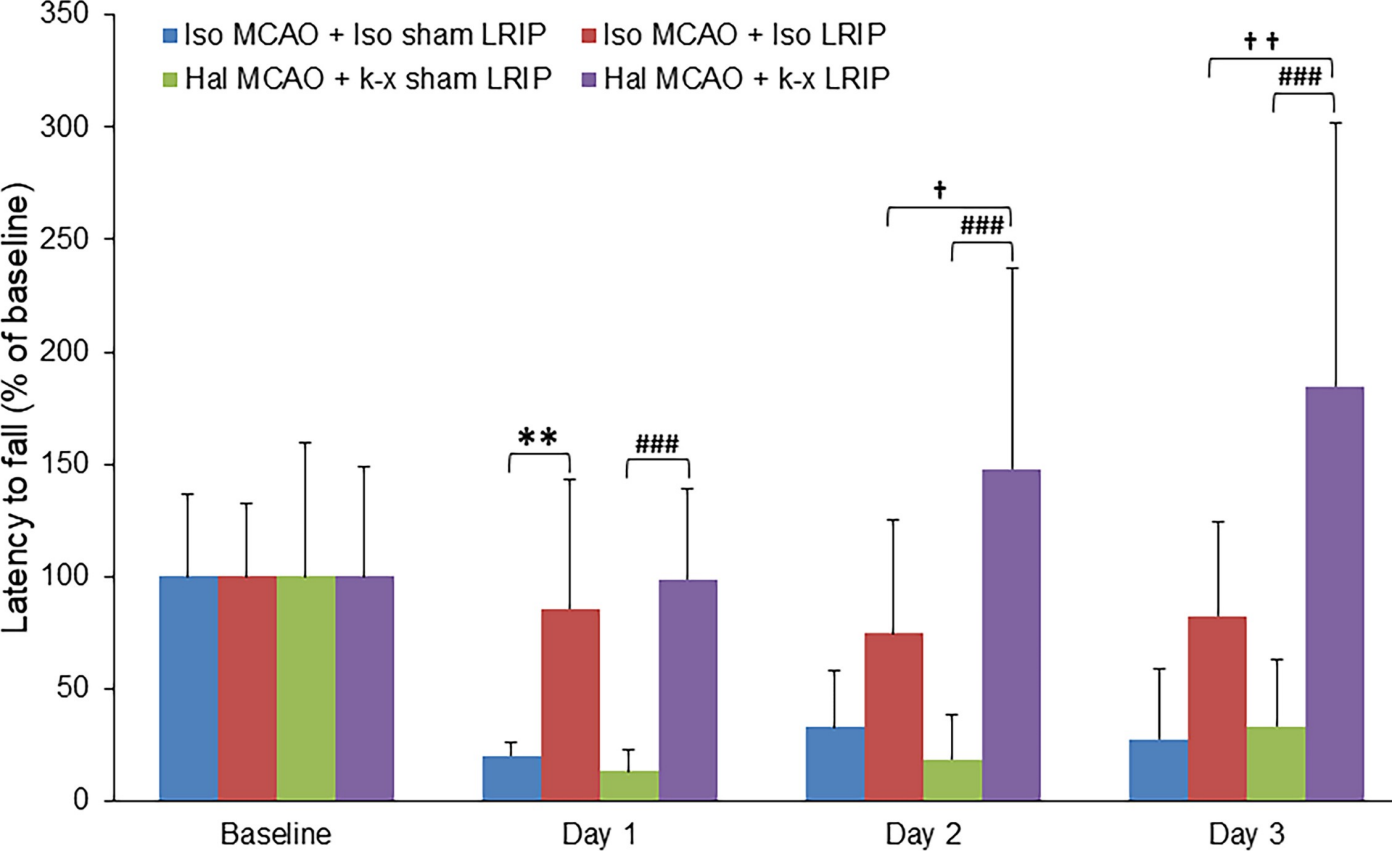
Effects of cerebral ischemia and LRIP on rotarod scores. The latency to fall from the moving rotarod was significantly attenuated by LRIP in MCAO. LRIP significantly improved the deficit and improved the latency to fall. Interestingly, latency to fall further improved significantly in cohort 4 compared with cohort 2. Data are presented as mean ± SD. **p < 0.01 when compared cohort 1 vs cohort 2, ^###^p < 0.001 when compared cohort 3 vs cohort 4, and ^†^p < 0.05, ^††^p < 0.01 when compared cohort 2 vs cohort 4.

LRIP treatment and the use of isoflurane significantly (p < 0.01) increased the duration of time on the rotarod as compared to the ischemic group on day 1, while there was a beneficial trend on day 2 and 3 post MCAO. Similarly, mice in cohort 4 using ketamine-xylazine in the LRIP treatment group spent a significantly (p < 0.001) greater amount of time on the rotarod than mice from cohort 3 on all 3 days post MCAO. Interestingly, there was also a significant difference between cohort 2 and cohort 4 mice at day 2 (p < 0.05) and day 3 (p < 0.01; Fig 3).

### Effect of LRIP and anesthetic on infarct volume

**Fig 4:**
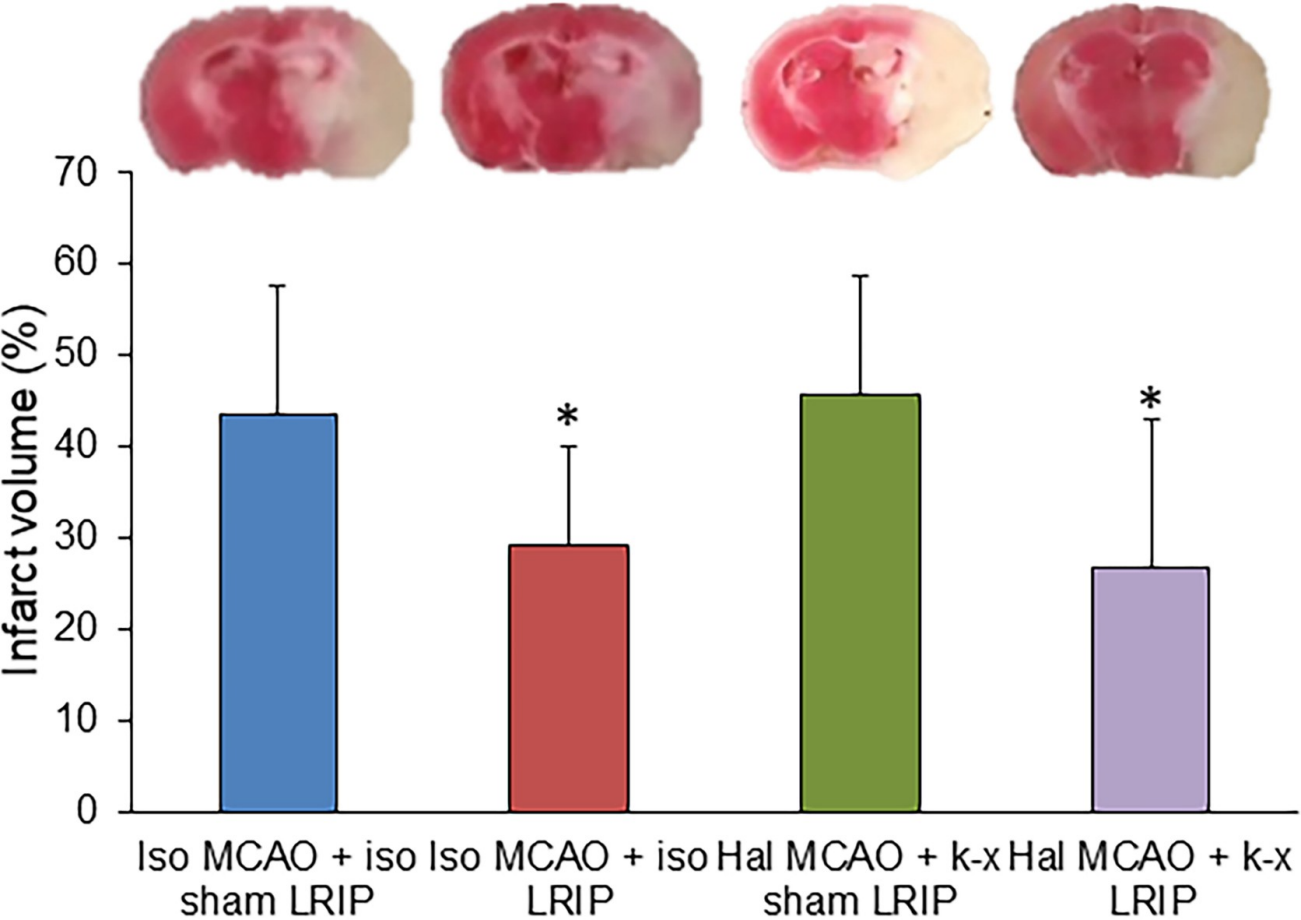
Effects of LRIP and anesthetic used on infarction volume after MCAO in mice. (A) The infarct area was observed by TTC staining of brain slices from each cohort. The viable tissue is stained red by TTC staining, but the infarcted area remains unstained, exhibiting pale yellow/white color. (B) Quantitative evaluation of percent infarction volume in different cohorts of mice was observed at 76 h after MCAO. The LRIP treatment group had significantly lower infarction volume compared with the sham group. The infarction volume was similar in cohort 2 and cohort 4, suggesting that the reduction in infarction volume was independent of the anesthetic used. Data are presented as mean ± SD. n = 6–11, *p < 0.05.

There was a significant decrease in infarction volume in cohorts that were subjected to LRIP after stroke, and this effect was independent of the anesthetic used during LRIP. However, there was no significant difference between cohorts 1 and 3, showing that the use of isoflurane or halothane during stroke had similar effects on infarction volume. Similarly, there was no difference between cohorts 2 and 4, showing that the beneficial effect observed by LRIP on infarction volume is independent of the anesthetic used (Fig 4).

## Discussion

In preclinical studies, the use of LRIP has been described as having beneficial effects on stroke outcomes. Moreover, the use of various anesthetics has been reported to have a paradoxical effect on stroke outcomes. As some commonly used anesthetics are also used during LRIP, we wanted to investigate whether these anesthetics have any adverse or confounding effects along with LRIP on stroke outcomes. Here, we used isoflurane or halothane at the time of MCAO surgery and isoflurane or ketamine-xylazine during LRIP. We found that LRIP significantly attenuated functional deficits and infarction volume, irrespective of the anesthetic used. Interestingly, the use of ketamine-xylazine during LRIP had a beneficial effect on functional outcomes than the use of isoflurane; however, there was no significant difference in infarction volume between the groups treated with either isoflurane or ketamine-xylazine during LRIP. Therefore, these data suggest that the choice of anesthetic to be used during LRIP should be carefully considered based on the expected outcomes.

Since isoflurane is one of the widely used anesthetics during various surgical procedures, we used it during MCAO surgery as well as during LRIP in cohorts 1 and 2. However, because isoflurane is also reported to have a protective effect on cerebral ischemia, to test if the effects observed in cohorts 1 and 2 could be due to isoflurane and not the LRIP, we used halothane to induce MCAO and ketamine-xylazine during LRIP in cohorts 3 and 4. Accordingly, we were able to compare the effect of LRIP under isoflurane and ketamine-xylazine and our data suggest that the use of ketamine-xylazine during LRIP provides beneficial effects in functional outcomes, although the impact on infarction volume was similar under both anesthetics (isoflurane and ketamine-xylazine). Similarly, we did not use ketamine-xylazine during MCAO because one of our exclusion criteria includes incomplete occlusion of the MCA, which is primarily determined by a change in CBF and secondarily by circling behavior during MCAO and after reperfusion. This testing is more precise when a gaseous anesthetic such as halothane or isoflurane is used because such anesthetics can cause animals to wake up a short time after the anesthesia is discontinued. With injectable anesthetics such as ketamine-xylazine, however, mice will take a longer time to wake up, and we may not be able to determine the circling behavior precisely.

It has been reported that LRIP effectively reduces the ischemic injury of the brain [34], heart [35], or other organs [36] in experimental and clinical research. Many anesthetics are reported to be able to reduce the ischemic brain injury by offering neuroprotection through anti-inflammatory [6] or anti-apoptotic/anti-cell death effects [8]. Apart from that, a growing body of preclinical literature also implicates anesthetic agents such as barbiturates, volatile anesthetics, propofol, and isoflurane in exhibiting neuroprotective properties in various preclinical studies [37,38]. Hence, using these anesthetics may affect the mechanisms related to neuroprotection offered by LRIP during cerebral ischemia. In this study, we also used halothane during MCAO surgery to compare the effect of isoflurane and halothane. Our data indicate that both anesthetics had a similar impact on infarction volume.

The body weight changes (as reported in Table 1) is one of the important consequences after ischemic stroke. The results of this study indicate that the body weight of each group decreased after MCAO, which is consistent with previously reported studies. However, no significant differences were observed among the groups. We also observed that LRIP, irrespective of the anesthetic used during LRIP, significantly decreased the neurologic deficit after stroke. Interestingly, the group where ketamine-xylazine was used during LRIP had significantly greater improvement in functional outcomes compared with the group where isoflurane was used during LRIP. Alteration in motor function is also an important aspect of ischemic brain injury; for this reason, we tested the motor function of mice by analyzing the latency to fall from an accelerating rotarod. The rotarod data suggest that the LRIP treatment in mice was able to extend the latency to fall from the rotarod. LRIP treatment improved motor function. Interestingly, we did not observe an increase in latency to fall by LRIP on day 1. This could be because the rotarod test is challenging for mice and that at 24 h after MCAO, they might still be too physically weak to take on this test. Nevertheless, these results indicate that the use of ketamine-xylazine during LRIP may have additional beneficial effects on neurologic functions; however, further studies are needed to understand the mechanism of action of ketamine-xylazine treatment.

**Table 1:** Changes in body weight across groups.

Groups	Pre-MCAO (g)	Post-MCAO (g)	Percent change in body weight
Iso MCAO + iso sham LRIP	25.5±1.5	23.1±1.2	9.5 ± 4.8
Iso MCAO + iso LRIP	25.7±1.2	23.4±0.9	9.0 ± 3.9
Hal MCAO + k-x sham LRIP	25.1±1.7	23.3±2.1	8.4 ± 6.9
Hal MCAO + k-x LRIP	25.3±1.5	22.9±1.5	9.4 ± 6.1

Cerebral infarction usually occurs after the blockage of the cerebral artery in the brain, which potentially affects the brain and neuronal function. To quantify the infarct volume, we performed TTC staining. TTC staining is one of the methods used to analyze the viable tissue, notably at earlier time points. From the TTC results, we found that LRIP was able to reduce the infarct volume after MCAO, irrespective of the use of isoflurane or ketamine-xylazine. This suggests that there was no effect of isoflurane or ketamine-xylazine use during LRIP on brain infarct volume. It is important to note that LRIP resulted in a significant decrease in functional deficits in cohort 4, where ketamine-xylazine was used during LRIP as compared with cohort 2, and where isoflurane was used during LRIP. However, there was no difference in infarction volume between cohorts 2 and 4. This raises the question of why we were not able to see similar disparities in functional and anatomical outcomes. It has been observed in various preclinical and clinical studies that often functional outcomes are not fully translated into anatomical betterment or vice versa [39,40]; however, molecular or physiologic events responsible for such discrepancies remain elusive. Based on such findings where there is a miss-match between functional and anatomical outcomes, recent attention has focused on improving the quality of life after stroke. This means that the focus should be on improving the functional outcomes or physical rehabilitation irrespective of whether anatomical outcomes are improved. Indeed, further studies are needed to fully understand why similar results were not observed for anatomical and functional outcomes in this study.

Some limitations of our study are that we have not investigated the neuroprotective mechanism of LRIP or how anesthetics may affect LRIP-related stroke outcomes. Although anesthetics are widely used during a range of procedures, their exact mechanism, and overall outcomes are still not completely understood. Some studies have suggested that isoflurane has some anti-cell death properties [41–43]. Ketamine is reported to reduce brain injury by inhibiting apoptosis [11,44,45] and exerting anti-inflammatory effects [6,46]. Therefore, we propose that the anesthetics used in this study, especially ketamine-xylazine, may affect the consequence of LRIP on stroke outcomes, partly through their anti-cell death properties and/or anti-inflammatory effects. Additional studies are needed to fully understand some of the mechanisms associated with LRIP-mediated neuroprotection and the interaction of LRIP with anesthetics.

In conclusion, our study suggests that the induction of LRIP is neuroprotective in ischemic mice and reduces the infarct area, motor function, and NDS. We have not observed any significant difference in infarct area when comparing isoflurane with ketamine-xylazine used during MCAO and LRIP, but there were differences in NDS and rotarod scores using these anesthetics. Hence, we conclude that LRIP confers neuroprotection against ischemic stroke, and the use of either ketamine-xylazine or isoflurane may affect the outcomes to some extent.

## Abbreviations

ECA: External carotid artery
LRIP: Limb remote ischemia post-conditioning
MCAO: Middle cerebral artery occlusion
MCA: Middle cerebral artery
NDS: Neurological deficit score
TTC: 2,3,5-triphenyhetrazolium chloride
